# Implementation of the ESC STEMI guidelines in female and elderly patients over a 20-year period in a large German registry

**DOI:** 10.1007/s00392-023-02165-9

**Published:** 2023-02-11

**Authors:** Leonhard Riehle, Raffaella M. Gothe, Jan Ebbinghaus, Birga Maier, Leonhard Bruch, Jens-Uwe Röhnisch, Helmut Schühlen, Andreas Fried, Martin Stockburger, Heinz Theres, Henryk Dreger, David M. Leistner, Ulf Landmesser, Georg M. Fröhlich

**Affiliations:** 1 Department of Cardiology, Charité - Universitätsmedizin Berlin, Corporate Member of Freie Universität Berlin and Humboldt Universität zu Berlin, Charitéplatz 1, 10117 Berlin, Germany; 2 Berlin-Brandenburger Herzinfarktregister, Berlin, Germany; 3 Department of Cardiology, Vivantes Humboldt-Klinikum, Berlin, Germany; 4 Department of Cardiology, Unfallkrankenhaus Berlin, Berlin, Germany; 5 Direktorat Klinische Forschung und Akademische Lehre, Vivantes Netzwerk für Gesundheit GmbH, Berlin, Germany; 6 Department of Cardiology, Klinik Nauen, Nauen, Germany; 7 Department of Cardiology, Martin-Luther Krankenhaus, Berlin, Germany; 8 Department of Cardiology, Vivantes Klinikum Kaulsdorf, Berlin, Germany

**Keywords:** ST-elevation myocardial infarction (STEMI), Women, Myocardial infarction, Coronary artery disease, Percutaneous coronary intervention (PCI)

## Abstract

**Aims:**

We investigated the implementation of new guidelines in ST-segment elevation myocardial infarction (STEMI) patients in a large real-world patient population in the metropolitan area of Berlin (Germany) over a 20-year period.

**Methods:**

From January 2000 to December 2019, a total of 25 792 patients were admitted with STEMI to one of the 34 member hospitals of the Berlin-Brandenburg Myocardial Infarction Registry (B2HIR) and were stratified for sex and age < 75 and ≥ 75 years.

**Results:**

The median age of women was 72 years (IQR 61–81) compared to 61 years in men (IQR 51–71). PCI treatment as a standard of care was implemented in men earlier than in women across all age groups. It took two years from the 2017 class IA ESC STEMI guideline recommendation to prefer the radial access route rather than femoral until > 60% of patients were treated accordingly. In 2019, less than 60% of elderly women were treated via a radial access. While the majority of patients < 75 years already received ticagrelor or prasugrel as antiplatelet agent in the year of the class IA ESC STEMI guideline recommendation in 2012, men ≥ 75 years lagged two years and women ≥ 75 three years behind. Amongst the elderly, in-hospital mortality was 22.6% (737) for women and 17.3% (523) for men (*p* < 0.001). In patients < 75 years fatal outcome was less likely with 7.2% (305) in women and 5.8% (833) in men (*p* < 0.001). After adjustment for confounding variables, female sex was an independent predictor of in-hospital mortality in patients ≥ 75 years (OR 1.37, 95% CI 1.12–1.68, *p* = 0.002), but not in patients < 75 years (*p* = 0.076).

**Conclusion:**

In-hospital mortality differs considerably by age and sex and remains highest in elderly patients and in particular in elderly females. In these patient groups, guideline recommended therapies were implemented with a significant delay.

**Graphical abstract:**

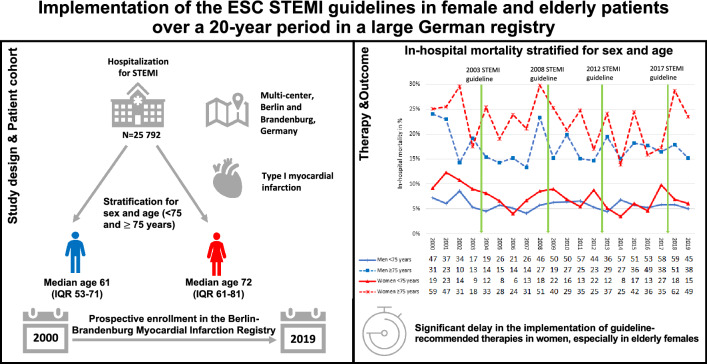

**Supplementary Information:**

The online version contains supplementary material available at 10.1007/s00392-023-02165-9.

## Introduction

ST-elevation myocardial infarction (STEMI) is associated with increased morbidity and mortality [1]. Nonetheless, several innovations and consecutive European Society of Cardiology (ESC) guideline updates were accompanied by a substantial reduction of in-hospital mortality that remains in the range from 4 to 12% in different ESC member countries in recent years [2]. Undoubtly, the main drivers for this improvement were the wide-spread use of revascularization therapy, primary percutaneous coronary intervention as a standard of care and modern antithrombotic agents [1, 3, 4].

Interestingly, the incidence of STEMI declined over the past three decades [5–7]. The main reasons for this phenomenon may be the substantial decrease in smoking prevalence, the wide spread use of statins and an improved awareness to monitor and treat hypertension as major cardiovascular risk factor [8, 9]. Despite these advances, STEMI remains a leading cause of death in western countries. [10, 11]

While the current ESC recommendations guide treatment in the majority of cases, several patient subsets need special consideration [12]. In particular, in elderly and female patients, guideline implementation might be delayed [13, 14].

Previous studies demonstrated higher in-hospital mortality and an increased risk of major adverse cardiovascular events (MACE) in females presenting with STEMI [15]. Elderly patients are more likely to receive palliation or conservative treatment for STEMI [16, 17]. Whether these disparities are largely due to the older age and higher burden of comorbidities of female STEMI patients remains unclear [18]. The most recent 2021 guidelines on Coronary Artery Revascularization, by the American College of Cardiology highlight the importance of continued vigilance against gender biases in the treatment of cardiovascular disease [19].

The aim of this study was to investigate the impact of ESC guidelines implementation on outcomes in different patient subgroups in a large prospective registry over the past two decades.

## Methods

### Data collection and patient population

The Berlin-Brandenburg myocardial infarction registry (B2HIR) was founded in 1999 to assess and improve the quality of care in patients presenting with acute coronary syndrome (ACS) at the participating member hospitals in Berlin and Brandenburg, Germany [20]. Details of the organization of the registry are described elsewhere [21]. The B2HIR prospectively collects data of patients with a type I myocardial infarction who are admitted within 24 h after the onset of symptoms [22]. In the present study, only patients presenting with STEMI according to the definition of the ESC guidelines were included [12].

The collected parameters were updated on a regular basis to reflect the continuous changes in the ESC guidelines and daily clinical practice. In the present study, 25,792 patients admitted to one of the member hospitals of the B2HIR between January 2000 and December 2019 were included in the analysis. Patients were stratified for sex and age < 75 and ≥ 75 years. Patients ≥ 75 years were defined as elderly as a majority of trials use 75 years as a cut-off value and mortality and prevalence of comorbidities sharply rise thereafter [12]. The member hospitals were responsible for data entry. Independent monitoring is performed and source-data are verified for an average of 7.5% of the registered patients. A data-plausibility check is performed annually for all member hospitals.

### Changes in the guidelines of the ESC in the time period of year 2000–2019

**Fig. 1:**
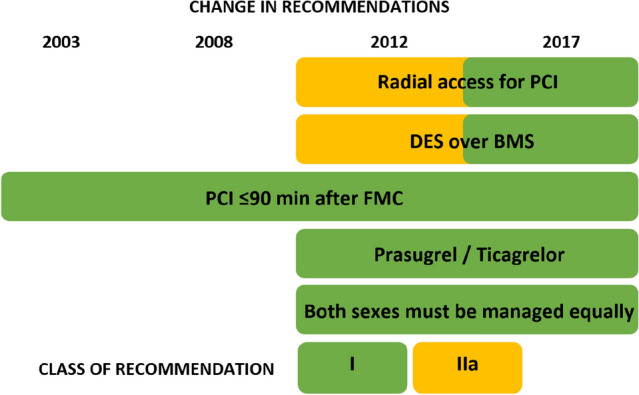
Major changes in recommendations for the treatment of STEMI in the ESC guidelines since 2003 ESC STEMI guideline [12, 24, 25, 47]. *PCI* percutaneous coronary intervention, *DES* drug-eluting stent, *BMS* bare-metal stent, *FMC* first medical contact

Over the study period, the European Society of Cardiology (ESC) issued four consecutive guideline updates for the diagnosis and therapy of STEMI in the years 2003, 2008, 2012 and 2017 [12, 23–25]. Figure 1 summarizes the major changes over the past 20 years.

### Outcome measures and endpoint definitions

The primary outcome measure of the present analysis was in-hospital mortality. Further outcome measures included a conservative versus an invasive approach, PCI and CABG rates, door-to-balloon time (DTB), the use of DES, radial access route for PCI, use of clopidogrel, ticagrelor and prasugrel as antiplatelet agents and MACE rates. MACE was defined as stroke, re-infarction, re-intervention, intervention-related bleeding (hemodynamically compromising or requiring transfusion), cardiopulmonary resuscitation, new onset of shock or need for mechanical ventilation and all-cause mortality. Major bleeding was defined as a hemodynamically compromising or requiring transfusion. Prehospital delay was defined as the time from symptom onset until hospital admission. Left ventricular ejection fraction as measured at time of admission using transthoracic echocardiogram. Successful guideline implementation as a standard of care of a new therapy (new antithrombotic drugs ticagrelor/prasugrel, drug eluting stents) or technique (radial access route) was arbitrarily assumed, if the innovation was applied in > 60% of patients per year.

### Statistics

Nominal data are presented as percentages and absolute numbers (parentheses), while continuous values are shown as median and interquartile range (1st and 3rd quartile). The Chi-square test was used to assess significant relationship between categorical variables, the Mann–Whitney *U* test was used to assess significant difference of continuous variables. Patients with missing values were excluded from the respective analysis.

In-hospital mortality of patients stratified by age (< 75 and ≥ 75 years) was analysed using a logistic regression model adjusted for the following variables: age, female sex, KILLIP IV or shock at admission, known heart failure, chronic kidney injury, smoking, diabetes mellitus, hypertension, prior myocardial infarction, prior PCI and atrial fibrillation at admission. Odds ratios (OR) and 95% confidence intervals (CI) were calculated.

The significance level was set to *p* < 0.05. Due to the exploratory nature of the analyses, no correction for multiple comparisons was performed. All analyses were performed using SPSS 25.0 (SPSS Inc., Chicago, USA).

### Ethics

The registry is approved by the Berlin Board for Data Privacy Monitoring. A positive ethics votum was granted by the Ärztekammer Berlin und Brandenburg. The study protocol confirms to the ethical guidelines of the 1975 Declaration of Helsinki [26].

## Results

### Baseline characteristics

A total of 25,659 patients were included into the study after exlusion of 260 patients with missing data on age and sex. Patients < 75 years were predominantly male (77.3%) while 51.8% of patients ≥ 75 years were female. The median age of men was 61 years (IQR 53–71) and 72 for women (IQR 61–81). The median age did not change significantly over the study period. Table 1 shows the baseline characteristics of male and female patients stratified by age. The median length of hospital stay declined from 11 days (IQR 16–8) for men and 14 days (IQR 19–10) for women to 5 days (IQR 7–3) for men and 6 days (IQR 8–4) for women from 2000 to 2019 (*p* < 0.001).

**Table 1:** Baseline characteristics for men and women stratified for age

	< 75 years					≥ 75 years				
	Men		Women		*p*	Men		Women		*p*
	*N*	(%)	*N*	(%)		*N*	(%)	*N*	(%)	
Median age (IQR)	14,845	58 (66–51)	4363	62 (69–54)	< 0.001	3108	79 (83–77)	3343	82 (68–78)	< 0.001
BMI < 18.5	130	1.0	95	2.6	< 0.001	43	1.7	109	4.2	< 0.001
18.5 to < 25	3616	29.1	1222	33.9	< 0.001	956	38.0	1065	41.2	< 0.001
25 to < 30	5799	46.7	1298	36.0	< 0.001	1156	46.0	973	37.6	< 0.001
≥ 30	2823	22.7	974	27.0	< 0.001	342	13.6	424	16.4	< 0.001
LVEF > 50%	3544	41.1	1059	42.5	< 0.018	478	27.3	531	31.4	< 0.013
41–50%	2034	23.6	532	21.3		383	21.8	341	20.2	
31–40%	906	10.5	297	11.9		253	14.4	251	14.8	
< 30%	609	7.1	148	5.9		252	14.4	189	11.2	
Initial HR > 100 bpm	1924	14.6	555	14.5	0.821	460	16.9	597	20.8	< 0.001
Smoker	8201	60.1	2167	53.2	< 0.001	478	17.9	321	11.1	0.014
Diabetes mellitus	2933	20.6	1060	25.2	< 0.001	964	32.7	1135	35.7	0.001
Hypertension	8911	63.4	2910	69.5	< 0.001	2394	81.0	2706	84.4	0.005
Hypercholesterinaemia	6459	48.2	1963	49.6	0.122	1284	47.1	1233	43.3	< 0.001
History of MI	2046	14.4	436	10.5	< 0.001	699	23.8	553	17.7	< 0.001
Pior PCI	2066	15.2	412	10.4	< 0.001	674	23.8	417	14.3	< 0.001
Prior CABG	412	3.0	72	1.8	< 0.001	229	8.0	99	3.3	< 0.001
History of stroke	354	3.5	107	3.7	0.603	194	8.9	193	9.7	0.399
Chronic HF	1316	9.3	412	9.9	0.245	675	23.3	740	23.8	0.615
Chronic KI	981	6.9	317	7.6	0.138	805	27.4	815	25.8	0.154
Atrial fibrillation	571	4.0	169	4.0	< 0.001	415	13.7	455	14.0	0.79
Pacemaker	78	0.6	35	0.9	0.037	61	2.2	48	1.7	0.165
KILLIP IV/shock	1342	10.1	385	10.0	0.073	389	13.8	359	12.3	0.86

*BMI* body mass index, *HR* heart rate, *MI* myocardial infarctiont, *PCI* percutaneous coronary intervention, *CABG* coronary artery bypass graft, *HF* heart failure, *KI* kidney injury, *LVEF* left ventricular ejection fraction (at admission)

### Prehospital delay and door-to-balloon time

Women, especially the elderly, presented with a significant delay to the emergency department after onset of ischaemic symptoms, if compared to male patients over the entire study period. In patients < 75 years, the median prehospital delay, defined as time from onset of pain to hospital admission, for men was 110 min. (IQR 66–242) compared to 122 min. for women (IQR 73–275, *p* < 0.001). This difference was even more pronounced in elderly patients ≥ 75 years with a median prehospital delay of 128 min. in men (IQR 75–291) compared to 158 min. (IQR 83–360) in women (*p* < 0.001). There was a marked improvement from year 2000 if compared to 2019 in the median prehospital delay times from 120 min. (IQR 76–240) to 102 min. (IQR 67–205) in men (*p* < 0.001) and from 150 min. (IQR 72–395) to 118 min. (IQR 76–250) in women (*p* < 0.001). However, time from symptom onset until hospital admission remains significantly higher in women in the year 2019 (*p* < 0.001).

**Fig. 2:**
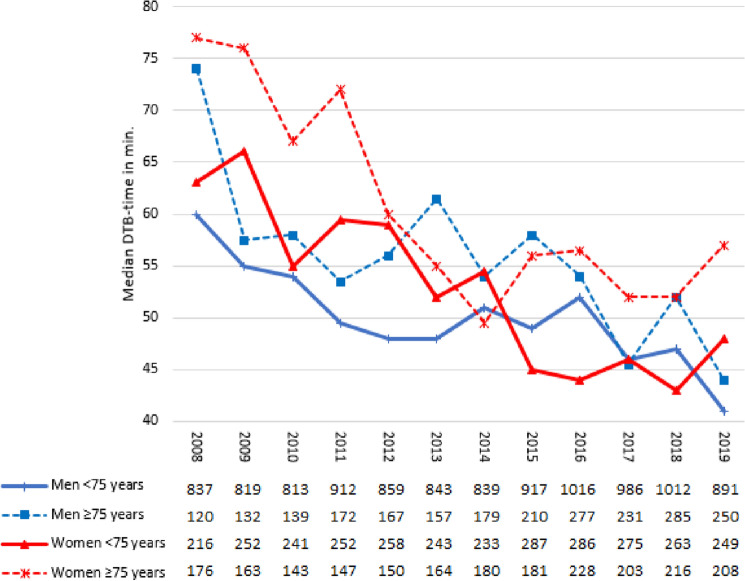
Median DTB in STEMI patients from 2008–2019 stratified for sex and age

The DTB-times (Fig. 2) decreased significantly from 62.5 min (IQR 30–115) in men and 67.0 min (IQR 40–152) in women in 2008 to 41 min (IQR 21–71) in men and to 49.5 min (IQR 27–96) in women in 2019 (*p* < 0.001 for both). In patients ≥ 75 years, these times decreased from 74 min (IQR 31–169) to 44 min (IQR 25–75) in men and from 77 min (IQR 49–191) to 57 min (IQR 27–133) in women (*p* < 0.001 for both). Until the end of the study period in 2019, the median DTB-time remained significantly longer in women compared to men, in particular in the elderly population over ≥ 75 years (*p* < 0.001).

### PCI rates and revascularization of non-culprit vessels during the index hospitalisation

**Fig. 3:**
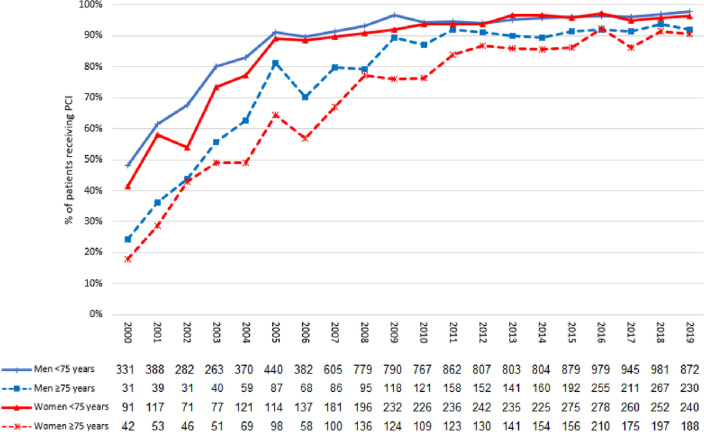
STEMI patients receiving PCI-therapy from 2000–2019 stratified for sex and age

Men < 75 years were more frequently treated with PCI compared to elderly women (89.9% (13,329) vs. 70.9% (2360), *p* < 0.001) over the entire study period (refer to Fig. 3). However, intervention rates increased continuously over the course of time. In the year 2000, 44.4% (362) of male and 29.4% (133) of female patients underwent PCI treatment (*p* < 0.001). In the year 2019, 97.9% (872) of male patients < 75 years were treated with PCI compared to 96.4% (240) of female patients < 75 years (*p* = 0.182). In the subset of patients ≥ 75 years, 92.0% (230) of male and 90.4% (188)of female patients received PCI treatment in the year 2019 (*p* = 0.542). PCI treatment was implemented as a standard of care (i.e., > 60% of patients) in men in 2001 compared to 2003 in women < 75 years. In the elderly, this threshold was only surpassed in the year 2004 for men and 2007 for women.

In 2018, 13.8% (37) of men (missing = 3) and 14.4% (28) of women above the age of 75 years and 11.8% (116) of men (missing = 3) vs. 8.4% (21) of women (missing = 1) below 75 years underwent a second PCI to a non-culprit vessel during the index hospitalization (*p* < 0.001).

In 2019 21.8% (40) of men (missing = 3) and 17.6% (33) of women above the age of 75 years and 10.4% (91) of men vs. 13.7% (33) of women below 75 years had a second intervention to a non-culprit vessel during the hospitalization (*p* < 0.001).

### Use of DES

DES were implemented as a standard of care in the majority of patients (> 60%) even before the class I recommendation of the 2017 STEMI guideline was published. In the first year of documentation of DES-use, less than < 60% of elderly men and women received DES while in patient group < 75 years, DES were already implemented as the standard of care. In the following year, > 60% of all patients received DES during PCI. In the year 2014, 92.6% (891) of male and 89.5% (342) of female patients (*p* = 0.284) received DES during PCI. In the following year 2015, no significant difference in DES-use in the different patient subsets was noted. From 2017 onwards, as DES were considered the new gold standard, the use of bare-metal or drug-eluting stents was no longer documented in the database.

### Antiplatelet therapy

Ticagrelor was increasingly used as an antiplatelet agent from 2010 onwards. Before the 2012 STEMI guideline, only 3.2% (27) of male and 2.4% (10) of female patients received ticagrelor in the year 2010. In 2019, there was no significant difference in ticagrelor use with 36.8% (468) of male vs. 39.8% (202) of female patients (*p* = 0.261). There was no significant difference in elderly patients. Prasugrel use is generally not recommended in patients ≥ 75 years or in patients with low body weight (< 60 kg). Consequently, prasugrel was less often used in female patients. In patients < 75 years, there was a significant difference in the use of prasugrel between men and women (57.3% (4416) vs. 53.3% (1154), *p* < 0.001). Across the entire study period, 50.9% (2296) of female patients received either ticagrelor or prasugrel compared to 55.3% (6372) of male patients (*p* < 0.001). In patients ≥ 75 years, clopidogrel remained the preferred antiplatelet agent for both men and women. 44.1% (774) of female patients received either ticagrelor or prasurel compared to 49.3% (934) of the male patients (*p* = 0.002) in this age group. However, after the class I recommendation in the 2012 STEMI guideline, > 60% of patients received either prasugrel or tricagrelor as an antiplatelet agent from the following year onwards.

### Radial access route for PCI

**Fig. 4:**
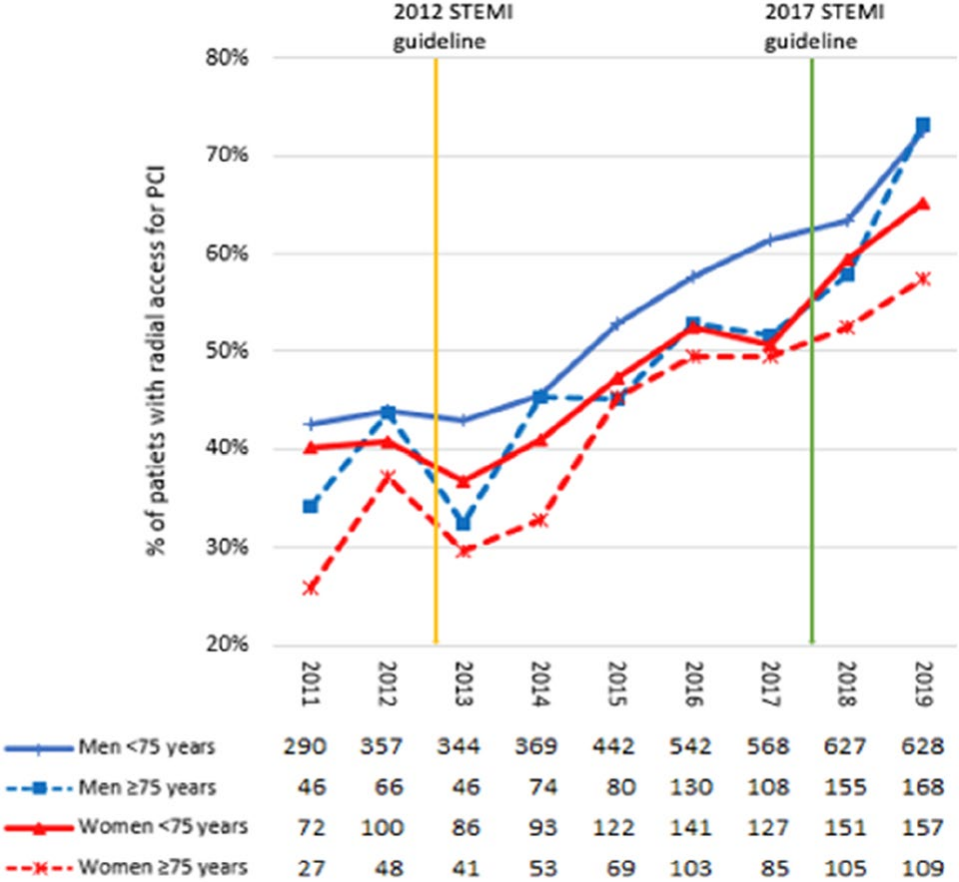
The radial access route for PCI from 2011 to 2019 stratified for sex and age

In 2011, radial access was used in 41.2% (336) of male and 35% (99) of female patients during PCI (*p* < 0.001) compared to 72.1% (796) male and 60.1% (266) female patients in 2019 (*p* < 0.001, Fig. 4). Notably, the radial access route was less common in elderly patients. In this subgroup, there was a significant difference in radial vs. femoral access between men and women (73.4% (168) vs. 57.4% (109) in 2019, *p* < 0.001). It took two years from the IA ESC STEMI guideline recommendation for preferred radial access until > 60% of patients were treated accordingly. In the year 2019, radial access was used in > 60% of all PCI-patients (Fig. 4). Only in the subset of patients < 75 years > 60% of men were treated radially in the year 2018 (Fig. 4).

MACE rates were significantly lower, if the transradial approach was used, if compared to the transfemoral route. In men < 75 years, MACE rates were significantly higher for the transfemoral access route with 15.3% (510) vs 5.6% (224), *p* < 0.001) compared to the transradial approach with similar results in women (16.9% (180) vs. 5.5% (55), *p* < 0.001). In elderly patients, 26% (204) of men treated via a transfemoral approach had an event, if compared to 12.8% (107) in the transradial approach (*p* < 0.001). In elderly women, this finding was even more pronounced the the transfemoral group with 29.6% (225) compared to 15.9% (92) in the transradial approach (*p* < 0.001).

### Major adverse cardiovascular events

Irrespective of age, in men with a BMI < 30 kg/m^2^, significantly more major bleeding events—defined as need for blood transfusion or leading to haemodynamic compromise—were observed [0.8% (78)], compared to obese men with a BMI > 30 kg/m^2^ (0.4% (10), *p* = 0.019). In women, there was a comparable difference between non-obese and obese patients that did, however, not reach statistical significance [1.6% (62) vs. 1.3% (16), *p* = 0.494]. However, bleeding events that did not trigger blood transfusions or significant haemodynamic compromise were not recorded in the present database.

MACE rates were significantly lower, if the transradial approach was used, if compared to the transfemoral route. In men < 75 years, MACE rates were significantly higher for the transfemoral access route with [15.3% (510) vs. 5.6% (224), *p* < 0.001] compared to the transradial approach with similar results in women [16.9% (180) vs. 5.5% (55), *p* < 0.001]. In elderly patients, 26% (204) of men treated via a transfemoral approach had an event, if compared to 12.8% (107) in the transradial approach (*p* < 0.001). In elderly women, this finding was even more pronounced the transfemoral group with 29.6% (225) compard to 15.9% (92) in the transradial approach (*p* < 0.001).

Across the entire patient population, the incidence of MACE declined from 16.8% (150) in 2008 to 12.7% (200) in 2019 (*p* < 0.001). In the subset of female patients ≥ 75 years, MACE rates were the highest with 26.4% (517) compared to 20.7% (441) in men ≥ 75 years. In patients < 75 years, MACE rates were lower with no statistically significant difference between men [10.3% (1030))] and women [11.6% (332), *p* = 0.053, refer to Table 2].

**Table 2:** MACE stratified for age and sex

	< 75 years					> = 75 years				
	Men		Women		*p*	Men		Women		*p*
	*N* = 9962	(%)	*N* = 2861	(%)		*N* = 2134	(%)	*N* = 1957	(%)	
Shock	154	1.5	28	0.9	0.025	52	2.3	76	3.7	0.008
Intubation	150	1.4	50	1.7	0.345	51	2.3	66	3.2	0.061
Reanimation	345	3.3	108	3.6	0.407	116	5.2	123	6.0	0.253
Reinfarction	192	1.3	69	1.6	0.007	58	1.9	90	2.8	< 0.001
Stroke	91	0.6	37	0.9	0.004	35	1.2	40	1.2	0.735
Reintervention due to ischaemia	237	1.9	69	1.9	0.964	52	2.0	41	1.6	0.275
Major bleeding	77	0.6	43	1.2	< 0.001	30	1.1	54	2.1	0.007
In-hospital mortality	833	5.8	305	7.2	< 0.001	523	17.3	737	22.6	< 0.001

### In-hospital mortality

In-hospital mortality decreased significantly over the study period from 12.8% (157) in 2000 to 9.2% (147) in 2019 (*p* < 0.001). Women had higher rates of in-hospital mortality in all age groups. Mortality declined from 24% (31) in the year 2000 to 15.2% (38) in the year 2019 (*p* = 0.035) in men over ≥ 75 years. In female patients ≥ 75 years, mortality rates remain at the highest levels of all patient subgroups [25.1% (59**)** in 2000 vs. 23.6% (49) in 2019, *p* = 0.705]. In patients < 75 years, the in-hospital mortality decreased over the study period and remained on a substantially lower level than in elderly patients. After adjustment for confounding variables, female sex was associated with an increased in-hospital mortality with an OR of 1.37 (95% CI 1.12–1.68, *p* = 0.002) in patients ≥ 75 years (Table 3). In patients < 75 years, female sex was not a significant predictor of in-hospital mortality (*p* = 0.076).

**Table 3:** Logistic regression for in-hospital mortality stratified for age

	< 75 years (*n* = 13,256)				≥ 75 years (*n* = 3994)			
	OR	95% CI		*p*	OR	95% CI		*p*
Female sex	1.229	0.98	1.54	0.076	1.370	1.12	1.68	0.002
Age, y	1.051	1.04	1.06	< 0.001	1.054	1.04	1.07	< 0.001
Diabetes mellitus	1.721	1.37	2.16	< 0.001	1.446	1.18	1.78	< 0.001
Hypertension	0.683	0.55	0.86	0.001	0.736	0.57	0.95	0.021
History of HF	1.254	0.96	1.65	0.102	1.480	1.18	1.86	< 0.001
Chronic KI	2.084	1.57	2.77	< 0.001	1.554	1.25	1.93	< 0.001
Current smoker	1.221	0.99	1.51	0.066	0.810	0.60	1.10	0.178
KILLIPIV or shock	17.434	14.17	21.45	< 0.001	10.272	8.09	13.04	< 0.001
Prior MI	1.184	0.81	1.74	0.387	1.167	0.86	1.58	0.316
Prior PCI	1.064	0.74	1.54	0.741	0.847	0.62	1.15	0.292
Afib at admission	1.245	0.87	1.79	0.237	1.198	0.92	1.56	0.184

*MI* myocardial infarctiont, *PCI* percutaneous coronary intervention, *HF* heart failure, *KI* kidney injury, *Afib* atrial fibrillation

## Discussion

Our study illustrates a continuous improvement in the treatment of STEMI patients over the past 20 years. Overall, in-hospital mortality rates declined from 12.8% in 2000 to 9.2% in 2019. It can be hypothesized that the the main drivers for this development were related to the increasing use of drug eluting stents, radial access, new antiplatelet therapies and the continuous reduction of the door-to-balloon-time [27–29]. Moreover, MACE rates decreased, possibly through the growing experience with the use of radial access route [30].

In-hospital mortality remained at a relatively high level in the analyzed real-world population. This finding may be explained by the high median age of female patients (72 years) and the high proportion of patients presenting with Killip class IV or cardiogenic shock (10.8%). Registries with comparable patient characteristics reported similar outcomes [31, 32].

Innovative medical and invasive therapies were continuously incorporated into ESC guideline updates. Nonetheless, the time delay to implement innovations into daily clinical practice varied widely. One year after the publication of the first landmark trial, radial access was rewarded a class IIA recommendation. Another five years later it received a class IA ESC STEMI guideline recommendation [12, 24, 33]. Finally, it took another two years until > 60% of patients were treated accordingly. We hypothesize that the switch from femoral to radial access as the default strategy was more challenging for the individual operator with a significant learning curve as compared to the use of DES or the change in antiplatelet drugs [34, 35]. This may be the main reason for the significant delay in using radial access in the majority of patients. In light of convincing evidence and the significant downsides of bare metal stents, DES were rapidly implemented into daily clinical practice a long time before the guidelines strongly supported their use (Table 4).

**Table 4:** Time from landmark-trial to Class IIA and Class IA recommendation in the ESC STEMI guidelines

	DES	Radial access	Modern antiplateled therapy
1st landmark trial	TYPHOO*N* (2006)	RIVAL (2011)	PLATO (2009)
Class IIA recommendation	2012 ESC	2012 ESC	n.a
Class IA recommendation	2017 ESC	2017 ESC	2012
> 60% of patients receiving guideline-based therapy^a^	2012	2019	2012

^a^either Ticagrelor or Prasugrel considered as guideline based antiplatetelet agents in patients without oral anticoagulation

In contrast, newer antiplatelet drugs, like ticagrelor or prasugrel, were rapidly embraced by the interventional community as the preferred treatment option. Only three years after publication of the first landmark trial the new platelet inhibitors received a class IA recommendation [12, 24, 36]. In the same year, these drugs were already the preferred treatment option in the majority of patients in the present analysis (Table 4).

Notably, all of these guideline changes were first implemented in younger —in particular male—patients. In elderly and female patients, all of the above mentioned innovations were introduced into daily clinical practice with a significant delay.

Compared to men, women presented with STEMI at an older age. In line with previously published data, we demonstrated that female STEMI patients are less likely to undergo revascularization in the presence of co-morbidities [37, 38]. The time delay from symptom onset to hospital admission as well as the door-to-balloon time (DTB) were significantly longer in women—and in particular in elderly females. Notably, the DTB time remained significantly longer in women than in men over the study period.

This observation may be associated to the poor in-hospital mortality in these patient groups (17.3% in men vs. 23.6% in women ≥ 75 years, *p* < 0.001). Similar results were published previously, demonstrating a two-fold in-hospital mortality in women as compared to men [39, 40]. Another possible explanation for the mortality difference might be an atypical presentation of females and older individuals that may postpone diagnosis and consequently revascularization. [31, 40, 41].

Similarly, radial access was used less frequently in women although the MATRIX trial showed greater benefit for PCI via the radial access route in women relative to men in terms of bleeding complications and even mortality [42].

Female sex appeared as an independent predictor of in-hospital mortality in patients ≥ 75 years. However, this finding may mainly be driven by an unfavorable risk profile along with age and baseline comorbidities [18, 43, 44]. Previous studies reported conflicting findings on the role of female sex as an independent predictor of in-hospital mortality which warrants further investigation of female-specific cardiovascular risk factors [40, 45, 46].

The present study is inherent to several limitations. Though data were collected prospectively, the data analysis was not pre-specified and therefore this analysis is retrospective in nature. While precise data on the course of disease were recorded during the index hospitalization, no follow-up data after hospital discharge were available. Due to data privacy concerns it was not possible to collect follow-up data on outcomes nor data on further coronary interventions over time. Moreover, parameters to describe the detailed coronary anatomy were not gathered in the present database. Data were collected exclusively in hospitals based in the metropolitan area of Berlin, Germany. The conclusions drawn by the presented data might, therefore, be only partly applicable to rural areas.

## Conclusion

Over the past 20 years, marked improvements in the treatment of STEMI were achieved. Importantly, in elderly patients and in particular in elderly females, new ESC guidelines were implemented into daily clinical practice with a significant time delay. Innovations that require a specific technical skillset with a consecutive learning curve need more time to gain popularity amongst operators. Future guidelines should, therefore, highlight the benefit of new recommendations in these traditionally undertreated patient subsets.

## Supplementary Information

Below is the link to the electronic supplementary material.

Supplementary file1 (DOCX 14 KB)

## Data Availability

The datasets generated and/or analysed during the current study are not publicly available due to German data protection regulations but are available from the corresponding author on reasonable request.
