# Supplementary material for: Implementation of the ESC STEMI guidelines in female and elderly patients over a 20-year period in a large German registry
Source: Clin Res Cardiol. 2023 Feb 11;112(9):1240–51. doi: 10.1007/s00392-023-02165-9 (PMC10449958; doi:10.1007/s00392-023-02165-9)
Supplement: Supplementary file 1 — Supplementary file1 (DOCX 14 KB) [file 392_2023_2165_MOESM1_ESM.docx]

**Supplementary Material Table 1. Logistic regression for in-hospital mortality for female patients only (N=4110)**

|  | OR | 95% CI | | p |
| --- | --- | --- | --- | --- |
| Age, y | 1.059 | 1.046 | 1.073 | <0.001 |
| Diabetes mellitus | 1.294 | 0.999 | 1.677 | 0.051 |
| Hypertension | 0.622 | 0.457 | 0.848 | 0.003 |
| History of HF | 1.219 | 0.907 | 1.637 | 0.189 |
| Chronic KI | 1.466 | 1.095 | 1.963 | 0.010 |
| Current smoker | 0.827 | 0.597 | 1.144 | 0.251 |
| KILLIPIV or shock | 2.539 | 2.298 | 2.805 | <0.001 |
| Prior MI | 1.074 | 0.693 | 1.663 | 0.751 |
| Prior PCI | 0.910 | 0.591 | 1.401 | 0.667 |
| Afib at admission | 1.243 | 0.864 | 1.790 | 0.241 |

*MI= myocardial infarction; PCI= percutaneous coronary intervention; HF= heart failure; KI= kidney injury; Afib= atrial fibrillation*
